# Transmission Attenuation Power Ratio Analysis of Flexible Electromagnetic Absorber Sheets Combined with a Metal Layer

**DOI:** 10.3390/ma11091612

**Published:** 2018-09-04

**Authors:** Jorge Victoria, Adrian Suarez, Jose Torres, Pedro A. Martinez, Antonio Alcarria, Julio Martos, Raimundo Garcia-Olcina, Jesus Soret, Steffen Muetsch, Alexander Gerfer

**Affiliations:** 1 Department of Electronic Engineering, University of Valencia, 46100 Burjassot, Spain; Jorge.Victoria@we-online.de (J.V.); Jose.Torres@uv.es (J.T.); Pedro.A.Martinez@uv.es (P.A.M.); Antonio.Alcarria@we-online.de (A.A.); Julio.Martos@uv.es (J.M.); Raimundo.Garcia@uv.es (R.G.-O.); Jesus.Soret@uv.es (J.S.); 2 Würth Elektronik eiSos GmbH & Co. KG, 74638 Waldenburg, Germany; Steffen.Muetsch@we-online.de (S.M.); Alexander.Gerfer@we-online.de (A.G.)

**Keywords:** flexible electromagnetic absorber sheet, complex permeability, magnetic materials, electromagnetic interference, power absorption, microstrip line, insertion loss

## Abstract

Electromagnetic noise absorber sheets have become a solution for solving complex electromagnetic interference (EMI) problems due to their high magnetic losses. This contribution is focused on characterizing a novel structure that is based on an absorber film with a metal layer attached on its top side. Two different absorber compositions were combined with Al and Cu metal layers in order to study the improvement on the performance of these structures, depending on the complex permeability, absorber film thickness, and type of metal. The transmission attenuation power ratio of the absorber films is analyzed and compared to the performance of absorber and metal structures. The measurement procedure is carried out attaching the films into a microstrip line that has been designed based on IEC standard (IEC 62333-2). This test fixture is employed as a transmission line to simulate a general noise path. The performance of absorber composites to filter electromagnetic noise is evaluated through analyzing *S*_21_ and *S*_11_ parameters. This is carried out with the aim of finding out in which conditions the absorption loss is improved when a metal layer is attached. In addition, the possible re-radiation effect, due to the magnetic field that is generated by the eddy currents induced in the metal layer, is examined.

## 1. Introduction

Electronic devices continually integrate more complex and more advanced functionalities. This involves the use of increasing operating frequencies, the miniaturization of the electronic design and high component integration, printed circuit board (PCB) size and thickness reduction, and using signals of very low voltage amplitude [1]. These design principles are often used to achieve a device with better performance and features; nevertheless, they increase the likelihood of generating complex electromagnetic interference (EMI) problems. The electronics can be sensitive to the surrounding electromagnetic environment, and it can also act as noise source, thus, it is very important to manage electromagnetic noise for avoiding unwanted electromagnetic interactions with nearby systems. Furthermore, at gigahertz (GHz) frequencies, semiconductor elements and PCB tracks radiate electromagnetic noise [2,3]. Therefore, the ability to control complex EMI problems either by eliminating or by reducing them is in high demand [4]. Consequently, electromagnetic shielding is one of the main concerns for electronic engineers, designing enclosures or devices that contain complex electronic systems [5]. 

Generally, the most common means of solving electromagnetic noise problems is to shield the system using a conductive shielded enclosure, foil tape, or conductive gasket. However, sometimes conductive shielding is not suitable due to size or weight restrictions and, thus, complex EMI problems cannot be resolved with conductive shielding techniques [6,7].

In this way, the use of noise suppression sheets (NSS) or flexible absorber sheets (FAS) has gained more interest in the solution of complex EMI problems because they are able to provide a lightweight solution with high flexibility and greater electromagnetic shielding than conductive shields [8,9]. These magnetic shielding materials have many advantages because they can be adapted to achieve complex shapes and are easily assembled with adhesive to a certain component or surface. Therefore, FAS represent a magnetic shielding solution with a great versatility in terms of mechanical properties.

Electromagnetic absorber materials are based on composite structures, usually a polymer host matrix with ferromagnetic metal flakes embedded [5,10,11]. Absorbing materials can be defined as a class of material with a specific ability to absorb and convert electromagnetic noise into heat. These types of amorphous magnetic thin films are classified in the categories of metamaterials, because they are able to provide electromagnetic properties that cannot be met by conventional homogeneous materials [1,12,13]. The capability that magnetic thin films present to reduce EMI is determined by its absorption loss and this depends on many factors, such as material parameters, frequency and sample dimensions [14,15]. Equation (1) [16,17] shows the influence of each last factor in order to determine the insertion loss (A), that is, the effectiveness of a certain shielding to filter electromagnetic noise. Thereby, the insertion loss depends on the relative permeability (${µ}_{r}$) and conductivity ($\sigma_{r}$) defined by the material composition, frequency (f), and the shield thickness (t):

(1) $$A=0.132t\sqrt{f{µ}_{r}\sigma_{r}}\;\mathrm{dB}$$

In this equation, the absorption loss or insertion loss A is expressed in decibel, t in millimeters, f in Hertz, and ${µ}_{r}$ and $\sigma_{r}$ are dimensionless parameters.

Many extensive studies on high frequency absorption properties of various materials have been carried out to look into how the parameters that interact in Equation (1) contributes to reduce the unwanted electromagnetic field. In fact, much attention has been paid in previous studies to analyze the conduction noise attenuation of magnetic thin films on transmission lines due to the different dominant factors, including: magnetic loss due to ferromagnetic resonance [3,18], dielectric loss provided for space charge polarization in the composites [19,20], and ohmic loss in electrically conductive sheets [21,22]. In particular, this contribution is focused on emulating electromagnetic conduction noise and evaluating two absorber sheets with the same thickness, shape, and size, but with different compositions. The main parameter studied is the transmission attenuation power ratio $\left(R_{tp}\right)$, since it is center on determining the attenuation of conducting current noise along electromagnetic noise paths achieve by the installation of a FAS by only measuring the S parameters. Thereby, $R_{tp}$ is used to characterize magnetic sheets and allow for obtaining the FAS attenuation ratio without needing to find out the material parameters and the thickness of the shield as occurs in the absorption loss general expression (Equation (1)). One of the sheets provides a higher magnetic loss value than the other in order to characterize how this difference could modify the performance of these FAS when a metal layer is placed on its top surface. The advantage of this multilayer shield structure mostly resides in the increase of the absorption loss due to the losses that were introduced by the metal layer. In this way, the metal layer can filter the unwanted electromagnetic field that is not absorbed by the magnetic film. This structure could extend the working bandwidth of these materials and obtain a greater attenuation than each of these materials could provide individually.

Thereby, different metals are combined with the two different FAS compositions with the aim of analyzing the effect of introducing a conductive plane on the absorber structure. There, the first shield, a high conductivity material, such as copper or aluminum, can provide a better reflection loss at higher frequencies than magnetic materials, whereas a magnetic film is a high-permeability material, which provides the maximum absorption loss at lower frequencies.

Because of the metal layers features, the re-radiation parameter is characterized to verify that the electromagnetic noise concentrated in its own metal layer does not generate an additional electromagnetic field that could act as an artificial noise source. Taking all of these issues into consideration, the $R_{tp}$ of different material combinations is determined and analyzed.

## 2. Materials and Methods

### 2.1. FAS Characterization

The flexible absorber sheet’s host matrix is based on a silicone rubber matrix and the inclusions embedded are iron particles in form of flakes prepared by the mechanical forging of spherical iron powders while using an attrition mill. When the size of the inclusions and the spatial periods are tiny compared with the wavelength of the electromagnetic field generated by the noise source, the composite materials can be considered as homogeneous materials [23,24]. Hence, this kind of material can be characterized through the geometrical and physical properties of the inclusions, the host medium, and the orientation of the inclusions regarding the host matrix. In addition to these characteristics, there are other properties that define the electromagnetic behavior: the volume fractions of the host material and inclusions, orientation and alignment of inclusions, statistical distribution of the parameters of inclusions, distance and contact between the inclusions, and the frequency dependence of constitutive parameters of the host material and inclusions [25,26].

Table 1 defines the characteristics of absorber sheets with 0.3 mm thickness in order to compare both compositions, although in the next section more sheet thicknesses will be employed in order to provide a wider material characterization.

**Figure 1:**
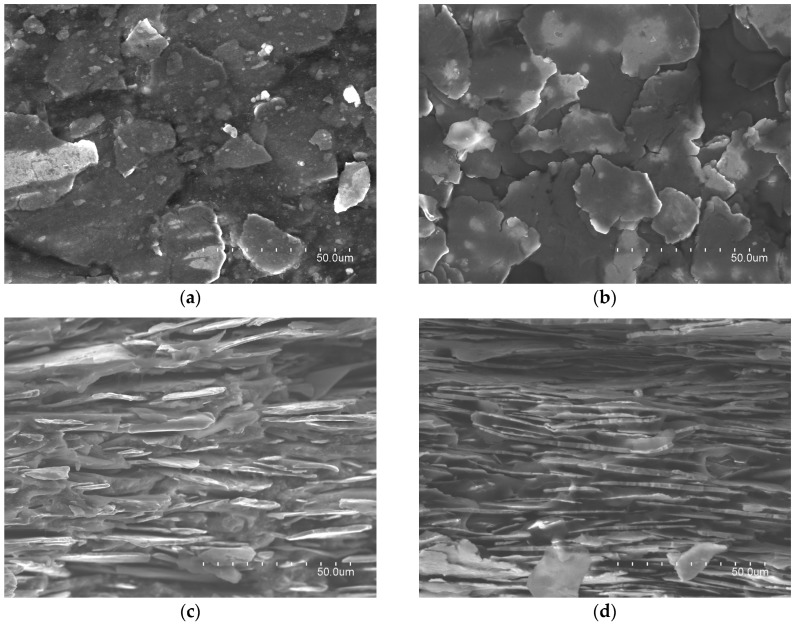
SEM photographs of electromagnetic absorber materials: (**a**) 304 material composition; (**b**) 3441 material composition; (**c**) cross-section of 304 material composition; and, (**d**) cross-section of 3441 material composition.

Figure 1 shows four micrographs of the samples, which were obtained with the S-4800 (Hitachi, Tokyo, Japan) scanning electron microscopy (SEM). The (a) and (b) photographs have been taken over the top surface of each material and they show the inclusions distributed into the host matrix. By comparing both images, it is possible to observe that 3441 material has a higher density of flakes, since it is difficult to distinguish gaps between them. On the other hand, 304 material shows a similar distribution, but in this case, the gaps between iron flakes can be identified because the density of this composition is lower. These gaps represent the polymer that joins the flakes. The in-plane orientation state of the flake in the FAS and the alignment of the inclusions regarding the host matrix can be observed in (c) and (d) micrographs, which show the FAS cross-section. These captions show how 3441 material has been manufactured to be more densely packed than 304 material. Thus, 3441 material integrates a higher number of flakes in the same sheet thickness, suggesting that this difference in its internal structure might be one of the most important factors why 3441 provides higher magnetic losses. 

These differences between both the characterized materials have also been detected by energy dispersive X-ray (EDX) to carry out the spectroscopy analysis with the S-4800 equipment. Both compositions are mostly based on iron oxide (${\mathrm{Fe}}_{2}O_{3}$) with a proportion of about 65% and 85% in the case of 304 and 3441 materials, respectively. This fact could imply that 3441 material shows a greater performance to absorb the electromagnetic noise.

One of the most important parameters that describe a material’s capacity to absorb electromagnetic noise is the relative permeability (${µ}_{r}$) [27]. The permeability relates the magnetic flux density to the magnetic field in a defined medium, thus, when an absorber sheet is placed, the magnetic flux is concentrated in it. This is defined by the composite material internal properties and it is represented as through the permeability complex parameter [28]. The losses of the magnetic flux can be quantified by separating it into its complex form, so that the real component (${µ}^{\prime }$) is related to the reflection or inductive part and the imaginary component (${µ}^{\prime \prime }$) quantifies the effectiveness of the material to absorb the magnetic noise [29]:

(2) $$\;{µ}_{r}={µ}^{\prime }-\;j{µ}^{\prime \prime }\;$$

The shielding performance to attenuate or suppress unwanted electromagnetic fields of composite structures can be achieved by the combination of the reflection and absorption losses [30]. The factors that define it are the conductivity, permeability, and thickness of the composite panel, as well as the frequency range of interest. The behavior of these parameters depends on the internal composition of the material. It is usually represented versus the frequency and it can be derived from electromagnetic theory [31,32] as well as obtained experimentally [33,34]. FAS suppress high-frequency noise, making use of the magnetic loss that is controllable according to the composition and shape of the magnetic material and it is related to the frequency domain separation. Thereby, in order to obtain significant electromagnetic noise attenuation at high-frequency region, it is necessary that the frequency of the ferromagnetic resonance is as high as possible [35]. 

**Figure 2:**
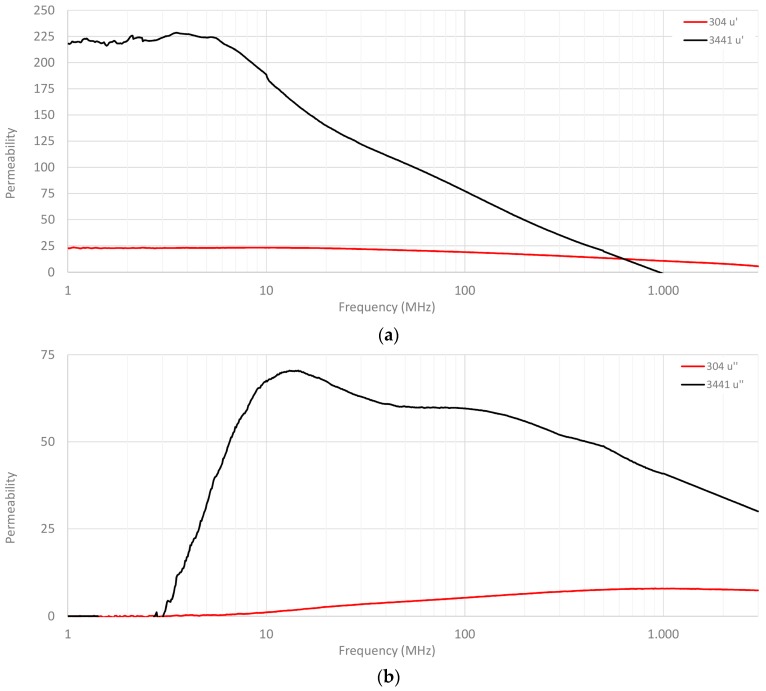
Complex relative permeability profiles of 304 and 3441 materials: (**a**) real part ${µ}^{\prime }$; and, (**b**) imaginary part ${µ}^{\prime \prime }$.

Therefore, one way of knowing the performance of 3441 and 304 materials is through examining the permeability parameter. Figure 2 represents the relative permeability profiles of 304 and 3441 FAS compositions split into real and imaginary components. As can be observed, 3441 material provided a higher initial permeability (${µ}_{i}$) than 304 composition, as has been described above, however, both compositions had a high relaxation frequency. Note that relaxation frequency ($f_{r}$) is an important parameter because it is the point where real and imaginary parts cross, and thus, the material changes its dominant behavior. The $f_{r}$ of 3441 material is 163 MHz, whereas the $f_{r}$ value of 304 material is 2.185 GHz. As can be observed in (b), 3441 composition provided a greater ${µ}^{\prime \prime }$ throughout the frequency range, that is, the ability to absorb electromagnetic noise. The results were obtained with an E4991A Material Analyzer (Keysight, Santa Rosa, CA, USA), together with the 16454A Magnetic Material Text Fixture. This fixture is based on cavity resonance and single-turn inductor theories and allows for measuring the magnetic properties of a material. The sample under test must have a toroidal shape in order to be able to fit inside the fixture, so that a toroidal core is manufactured with the material of each absorber composition. This toroidal sample has an outer diameter of 20 mm, 8 mm of inner diameter, and the thickness is 1 mm. When the sample is placed inside the fixture, an ideal single-turn inductor with no flux leakage is formed; therefore, the permeability can be calculated automatically by the internal software of the equipment from the inductance value of the sample. Thereby, it is possible to obtain direct complex permeability readouts.

### 2.2. Metal Layer Characteristics

Once the FASs had been analyzed and characterized, aluminum and copper metal layers, which were combined with two different absorber compositions, are described in order to study the improvement on the performance of these structures. The metal layers consisted of EMI shielding tapes that were fabricated of a soft aluminum or copper foil backing with an electrically conductive acrylic adhesive. These kinds of materials are usually used for applications that require a great electrical conductivity from the application substrate through the adhesive to the metal surface. Foil tapes with the same dimensions of absorber sheets were used in this study with the aim of covering them completely. In the case of aluminum foil, the surface resistance $R_{s}$ = 0.001 Ω/sq and for copper foil, $R_{s}$ = 0.0005 Ω/sq. Both shielding tapes have a thickness H = 0.04 mm.

### 2.3. Transmission Attenuation Power Ratio Measurement

Generally, these materials are applied in areas that are very close to the radiation source, thus knowledge of their behavior in the near field region becomes more relevant [1]. This contribution is focused on evaluating the performance of structures based on absorber sheets combined with a metal layer to reduce or suppress conduction electromagnetic noise in the near field range. Consequently, the transmission attenuation power ratio ($R_{tp}$) was determined following the procedure that is defined in the standard IEC 62333-2 [36] to evaluate the frequency profiles of electromagnetic absorber sheets by using a microstrip line (MSL). The MSL was used as a test fixture for evaluating the attenuation of conducting current noise in a PCB or noise path when a noise suppression sheet is installed. Thereby, the MSL was employed as a transmission line that provides the electromagnetic noise that will be measured to know the sample absorption capability [37].

**Figure 3:**
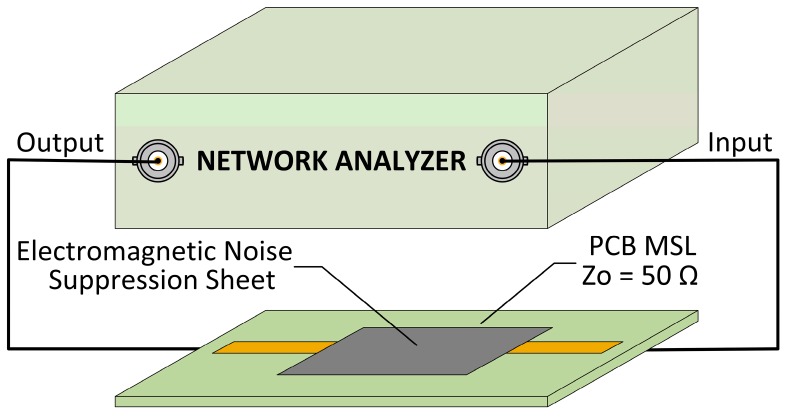
Measurement of reflection and transmission loss through microstrip line (MSL) test fixture.

The standard manufactured MSL with a characteristic impedance of 50 Ω was employed in this procedure. The fabricated MSL was based on the characteristics that are described in the standard due to Teflon PTFE substrate with dimensions of 100.0 mm length, 50.0 mm width and 1.6 mm thickness is employed. It consisted of a two layers PCB, where, on the top side, the copper strip conductor with 54.4 mm length, 4.4 mm width, and 0.018 mm thickness is printed. On the bottom side, a ground plane of copper with 0.018 mm thickness is placed and two SMA type connectors are connected are connected one at each end of the strip conductor. The SMA connectors were placed on the bottom layer and they were connected with both ends of the MSL through two vias. These vias are based on a copper conductor that joins different PCB layers; in this case, they are used to join the center conductor of each SMA connector with each end of the strip conductor. The MSL text fixture was used to determine the electromagnetic noise absorbing capability of a certain magnetic film by measuring the reflection and transmission parameters ($S_{11}$ and $S_{21}$, respectively) with an E5071B Network Analyzer (NA) equipment (Keysight, Santa Rosa, CA, USA), as shown in Figure 3. 

Once both $S_{11}$ and $S_{21}$ measurements were done, it was possible to calculate the $R_{tp}$ value expressed in decibel units using the equation defined by the standard IEC 62333-2:

(3) $$R_{tp}=-10\log\left(\frac{10^{S_{21/10}}}{1-10^{S_{11/10}}}\right)\;\mathrm{dB}$$

where *S*_11_ is the reflection coefficient, *S*_21_ is the transmission coefficient, and both parameters are magnitudes in decibels.

The measurement of the MSL transmission characteristics while using a NA made it possible to obtain transmission loss that is associated with magnetic loss of the magnetic material placed on the strip conductor. When a FAS is placed on the transmission line with conducted electromagnetic noise, the high-frequency current is reduced by an additional resistance due to the magnetic dynamic loss [15,38].

### 2.4. Re-Radiation Measurement Setup

**Figure 4:**
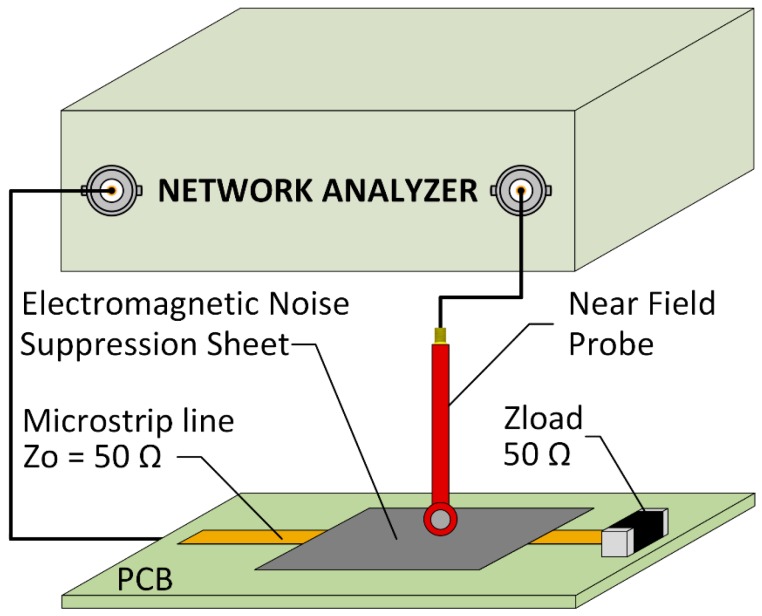
Re-radiation measurement setup.

The integration of a metal layer on the absorber sheet could lead to a re-radiation effect, because the electromagnetic noise concentrated into the metal could generate eddy currents that induce a parasitic magnetic flux [39]. Thereby, the measurement of samples re-radiation is presented in the next section with the aim of ensuring they are not emitting an unwanted electromagnetic field. The experimental procedure carried out to evaluate whether the re-radiation parameter was also based on using the MSL as an electromagnetic noise source and the NA equipment, however, in this case, one SMA connector of the MSL was connected to the NA and the other is terminated with a 50 Ω load. A near field probe (NFP) was connected to the second port of the NA and was placed over the center of the strip conductor forming a right angle with it as shown in Figure 4. Subsequently, the $S_{21}$ parameter was evaluated through only measuring the radiated energy by the MSL with the measurement being repeated with the absorber structure placed on the MSL. The distance was set at 1 mm from the strip line to the NFP. Finally, both $S_{21}$ parameters were compared in order to determine if the absorber structure was generating a re-radiation effect. 

## 3. Results and Discussion

Firstly, the transmission attenuation power ratio of both flexible absorber materials under test has been determined accordingly with the IEC standard method. Subsequently, this parameter was also obtained for the structures that are based on an absorber sheet with a metal layer added on its top side in order to analyze the improvement on the performance of these structures, depending on the complex permeability, absorber film thickness, and type of metal. When considering this, it was possible to evaluate the performance of a high permeability absorber material to provide a solution to filter the conducted electromagnetic noise in the range frequency of 1 MHz to 8.5 GHz and compare it to another material with lower magnetic loss.

### 3.1. $R_{tp}$ Depending on the Material Composition and Kind of Metal Layer

**Figure 5:**
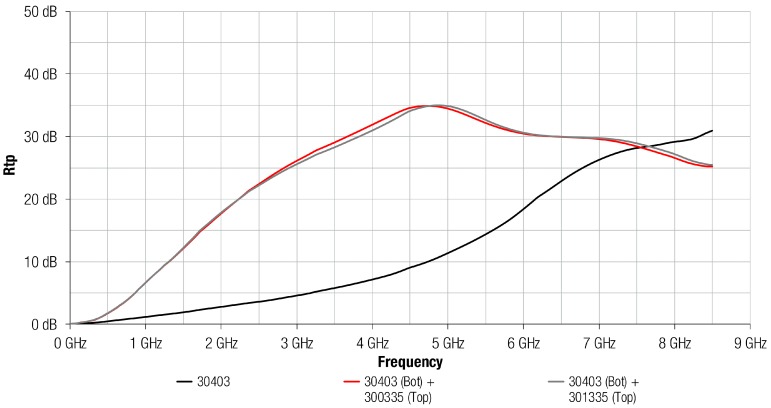
$R_{tp}$ of 30403 absorber sheet without any metal layer (black), 30403 absorber sheet with a Cu layer attached (red) and 30403 absorber sheet with a Al layer attached (grey).

As has been described in Section 2.3, the transmission attenuation power ratio was determined following the IEC 62333-2 standard based on a microstrip line fixture that simulates a source of electromagnetic conducted interferences. Figure 5 shows the $R_{tp}$ parameter measured when the 304 absorber material with a thickness of 0.3 mm was attached on the microstrip line. The $R_{tp}$ profiles of the structures based on the combination of 304 magnetic film with a Cu layer and Al layer are also represented. This graph demonstrates the ability of the material to attenuate the electromagnetic noise when a metal layer is combined with the 304 absorber composition, being higher than when only the magnetic film is used. Both magnetic and metal structures provided a similar performance, suggesting the difference between combining an Al or a Cu layer with the 304 absorber material is negligible. The structures that combine a metal with an absorber material reached the maximum value about 5 GHz, and, from that frequency, $R_{tp}$ decreased slowly. Nevertheless, 304 profile continuously increased throughout the frequency spectrum analyzed and provided a greater attenuation than the metal and magnetic absorber structures from 7.5 GHz. Note that the maximum difference of $R_{tp}$ between the absorber sheet 304 and both structures based on 304 material with a metal layer attached was about 25 dB and it is taken at 4.8 GHz. 

**Figure 6:**
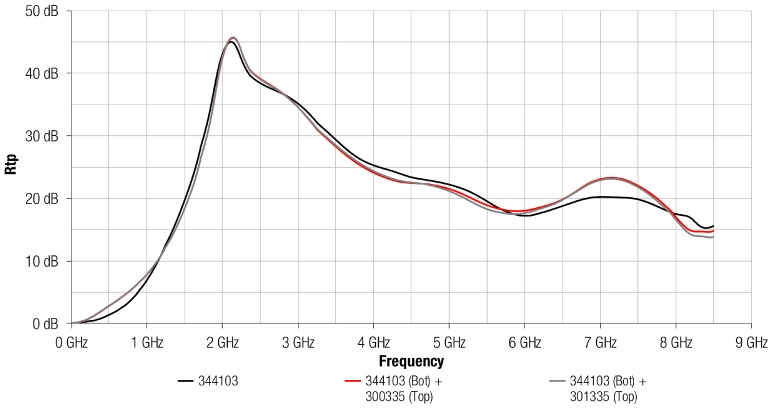
$R_{tp}$ of 344103 absorber sheet without any metal layer (black), 344103 absorber sheet with a Cu layer attached (red,) and 344103 absorber sheet with a Al layer attached (grey).

This measurement has been repeated, but in this case, using the 3441 magnetic film that provides a higher permeability than the 304 composition. Figure 6 shows the $R_{tp}$ parameter measured when the 3441 absorber material with a thickness of 0.3 mm was attached on the MSL and when it is combined with a Cu or Al layer. If the 3441 profile is compared to the absorption ability that is provided by 304 composition, it might be concluded that 3441 shows a greater attenuation than 304 up to 6 GHz. Due to its higher permeability, 3441 composition was able to provide a very high attenuation at lower frequencies than 304, reaching the maximum $R_{tp}$ close to 6 GHz. This material provided an abrupt increase at low frequencies, rising to 45 dB from 0 GHz to 2 GHz. From 2 GHz, 3441 trace decreased gradually, providing a higher value of $R_{tp}$ up to 6 GHz than 304 composition. With regard to the effect of placing an Al or a Cu layer on the 3441 magnetic film, the difference between using a copper layer or aluminum layer were negligibly low, similarly to when a metal layer was combined with 304 absorber sheet, as shown in Figure 5. Another factor that is important to highlight from Figure 6 is that all traces show a similar profile in contrast to Figure 5 where structures based on metal and 304 magnetic film provided a different performance from the 304 composition. This suggests that the performance of 3441 material is not improved when a metal layer is attached on it because of the higher magnetic losses given by 3441 magnetic material.

### 3.2. $R_{tp}$ Depending on the Material Composition and the Absorber Sheet Thickness

**Figure 7:**
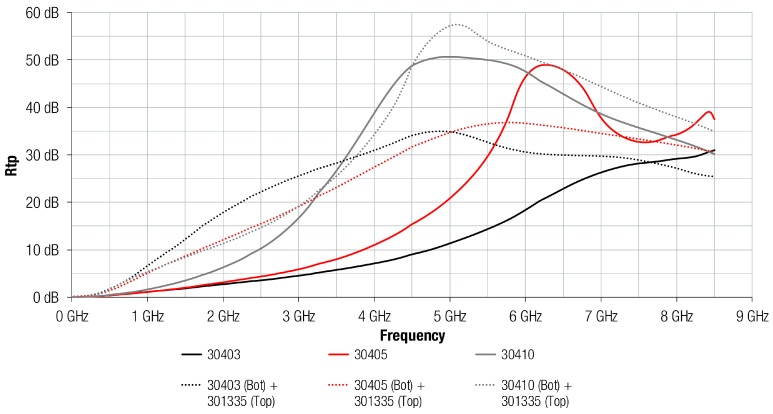
$R_{tp}$ of 304 absorber material with different thicknesses and Al layer integrated.

As can be observed from data shown in the last subsection, 304 and 3441 electromagnetic absorber materials did not provide the same behavior when combined with a metal layer. Thereby, 304 composition was analyzed depending on its sheet thickness and combining it with an Al layer, as shown in Figure 7. This graph demonstrates how the greater the thickness of the FAS, generally, the greater the attenuation offered. As regards magnetic films, the maximum $R_{tp}$ value was about 50 dB and was provided by 30405 (0.5 mm thickness) and 30410 (1.0 mm thickness) at 6.3 GHz and 5.0 GHz, respectively. Thus, materials with the same composition were able to offer the same maximum value of attenuation, although when the sheet thickness was increased, and, therefore, the magnetic loss, the maximum peak appeared at lower frequencies. The maximum difference between FAS with metal structures and FAS was 23 dB at 4.8 GHz, in the case of 30403 material; 13 dB at 3.0 GHz for 30405; and, 4.7 dB at 5.0 GHz for 30410. These results suggest that the improvement on the FAS performance with the metal layer attached is more significant when the FAS thickness is lower. Note also that the presence of the metal layer on the absorber generally improved its attenuation performance at lower frequencies. 

**Figure 8:**
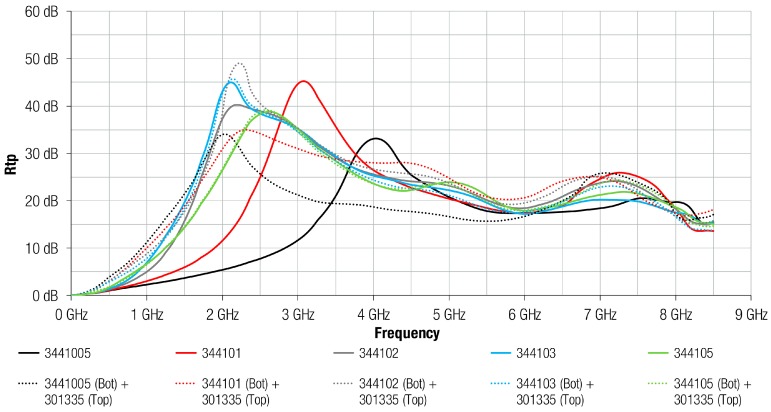
$R_{tp}$ of 3441 absorber material with different thicknesses and Al layer integrated.

Figure 8 shows the same comparison, between FAS and FAS with metal structures, as the previous graph, but in this case, the FAS material analyzed corresponds to the 3441 composition. It can be observed how the difference between FAS and FAS with Al structures is significant in the case of thinner sheets: 3441005 (0.05 mm) and 344101 (0.1 mm). Similar to 304 composition, the presence of the metal layer attached on the absorber improved the attenuation performance at lower frequencies for 3441005 and 344101. Specifically, for 344102, the traces were very similar, but the attachment of the metal layer on the absorber improved the maximum peak of $R_{tp}$ provided by 10 dB. However, for the 3441 composition the difference between FAS and FAS with Al structures was practically negligible for sheets with thickness higher than 0.2 mm. Because the 3441 material offers greater magnetic characteristics than 304 material, the effect of placing the metal layer was less significant than 304 in lower sheet thickness. In the case of 304, the effect of metal layer was negligible above 0.5 mm sheet thickness, whereas for the 3441 composition this happened above 0.3 mm sheet thickness.

### 3.3. Re-Radiation Analysis

**Figure 9:**
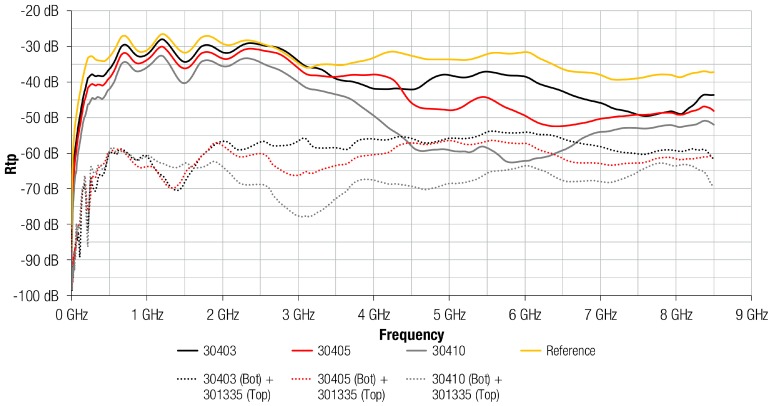
Re-radiation parameter of 304 absorber material with different thicknesses and Al layer integrated.

The results of the 304 composition re-radiation based on the measurement setup described in Section 2.4 are shown in Figure 9. From the figure, it is obvious that the ratio of re-radiation from the FAS with metal structure was negligibly low, because, in all thicknesses, when the Al layer was attached on the FAS, the radiated emissions were reduced. For instance, the 30403 composition is similar to the reference (yellow trace) electromagnetic noise measured without any sample placed on the MSL up 3 GHz. From this frequency point, the difference between re-radiation reference trace and re-radiation with 30403 magnetic film is increasing. On the other hand, the structure of 30403 FAS and Al layer reduced the re-radiation ratio by about 30 dB up to 3 GHz and by about 10 dB at higher frequencies. 

**Figure 10:**
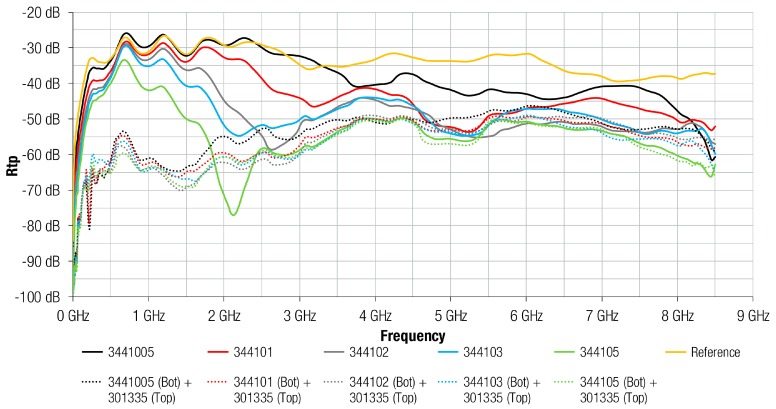
Re-radiation parameter of 3441 absorber material with different thicknesses and Al layer integrated.

Figure 10 shows the same comparison as Figure 9, but in this case, 3441 composition is evaluated in terms of re-radiation. The results suggest the same conclusion obtained for 304 material because all the traces show a re-radiation value lower than the reference trace measured without any sample on the MSL. Generally, when the Al layer was combined with the FAS, the re-radiation was reduced. When comparing the 3441 0.3 mm samples, the attenuation of FAS sample was higher from 1 GHz than 304 composition. When the Al layer was attached, this reduction is about 30 dB up to 3 GHz and about 10 dB at higher frequencies.

## 4. Conclusions

The noise absorbing properties of two kinds of flexible absorber sheets were analyzed in order to study the improvement on their performance when they are combined with a metal layer. Copper and aluminum layers were combined with both absorber materials and similar results were obtained, so that Al layer were selected to carry out more tests due to it usually representing a more common material and it offers a more competitive cost than Cu.

In terms of $R_{tp}$, 3441 composition provides a higher performance in the whole frequency spectrum because of it provides a higher magnetic loss than 304 composition. This result matches the relative permeability profiles that are provided for each material. Thus, it is also observed that the higher the thickness of absorber sheet, the higher the attenuation. With regard to the attachment of a metal layer on the absorber material, the results show that the improvement on the performance is more significant in the absorber materials with low magnetic loss. It has been observed when the metal layer has been added in the 30403 material due to the $R_{tp}$ has been increased about 25 dB in the maximum point. Nevertheless, it has not been observed any change when the high-permeability 344103 sheet with a metal layer attached has been analyzed.

Another conclusion that was obtained from the results, is related to the effect of placing a metal layer depending on the thickness of the absorber sheet. It has been proved that an absorber sheet with low thickness shows a greater $R_{tp}$ when the metal sheet is added. This suggests that the performance of absorbers with higher magnetic loss and thickness is not improved when the metal layer is integrated. This demonstrates that the improvement on the performance is negligible when a conductive layer is placed on a thicker absorber sheet, since the magnetic loss that is provided by the absorber is much more significant than the losses provided by the metal layer. Thus, the use of a metal layer provides a higher shielding effectiveness when it is attached to those sheets with low thickness. Accordingly, with this last point, the use of FAS and metal structure makes it possible to save space at the same time that provides high absorption loss without resorting to high-thickness absorber sheets.

Finally, the re-radiation parameter has been analyzed with the aim of ensuring that the presence of a metal layer on the absorber material does not lead to inducing a parasitic magnetic flux. It has been determined that placing the FAS and metal structure, not only it does not re-radiate, but also this combination decreases largely the level of electromagnetic radiated emissions measured in the near field. This is because the isolation is greatly increased when the metal layer is attached on the absorber sheet. Therefore, this opens the way for using the FAS and metal combination in applications with problems that are related to magnetic decoupling.

## Figures and Tables

**Table 1:** Comparison of magnetic properties for 3441 and 304 magnetic films.

Parameters	3441	304
Thickness (mm)	0.3	0.3
Size (mm)	50 × 100	50 × 100
Surface resistivity (Ω/sq)	1 × 10^6^	1 × 10^9^
Density (g/cm^3^)	3.9	3.5
Relaxation frequ. (GHz)	0.163	2.185
Initial permeability	200	25
